# Investigating the structural properties of amyloid-like fibrils formed *in vitro* from *β*_2_-microglobulin using limited proteolysis and electrospray ionisation mass spectrometry

**DOI:** 10.1002/rcm.2482

**Published:** 2006-01-01

**Authors:** Sarah L. Myers, Neil H. Thomson, Sheena E. Radford, Alison E. Ashcroft

**Affiliations:** Astbury Centre for Structural Molecular Biology, Astbury & Garstang Buildings, University of Leeds, Leeds LS2 9JT, UK

## Abstract

The protein *β*_2_-microglobulin (*β*_2_m) aggregates to form classical amyloid fibrils in patients undergoing long-term haemodialysis. Amyloid-like fibrils with a cross-*β* fold can also be formed from wild-type *β*_2_m under acidic conditions *in vitro*. The morphology of such fibrils depends critically on the conditions used: incubation of *β*_2_m in low ionic strength buffers at pH 2.5 results in the formation of long (μm), straight fibrils while, at pH 3.6, short (<500 nm) fibrils form. At higher ionic strengths (0.2–0.4 M) at pH 1.5–3.6, the fibrils have a distinct curved and nodular morphology. To determine the conformational properties of *β*_2_m within *in vitro* fibrils of different morphologies, limited proteolysis of each fibril type using pepsin was performed and the resulting peptide fragments identified by tandem mass spectrometry. For comparison, the proteolytic degradation patterns of monomeric *β*_2_m and seven synthetic peptides spanning the entire sequence of the intact protein were similarly analysed. The results show that fibrils with different morphologies result in distinct digestion patterns. While the curved, worm-like fibrils are relatively weakly protected from proteolysis, the long, straight fibrils formed at pH 2.5 at low ionic strength show only a single cut-site at Val9, demonstrating that substantial refolding of the initially acid-denatured and unprotected state of *β*_2_m occurs during assembly. The data demonstrate that the organisation of the polypeptide chain in fibrils with different morphological features differs considerably, despite the fact that the fibrils possess a common cross-*β* architecture.

Amyloid and amyloid-related diseases result in the formation of insoluble fibrils that deposit, usually with high specificity, in different locations in the human body.^1^ One of the initiating events of protein assembly into amyloid fibrils involves the complete or partial unfolding of the native protein to form aggregation-prone amyloid precursor states.^2,3^ Regardless of their initial structure, many proteins can be induced to form amyloid-like fibrils *in vitro* by judicious choice of the solution conditions that favour partial or complete unfolding of the polypeptide chain before intermolecular assembly.^4,5^ Despite the wide range of proteins that form amyloid fibrils *in vitro*, the fibrils generated all contain a common cross-*β* structure akin to that of fibrils formed in vivo, in which the core of the macromolecular structure is comprised of *β*-strands oriented perpendicular to the long axis of the fibril.^6^ This structure gives rise to a characteristic fibre diffraction pattern, together with a generic ability of amyloid fibrils to bind hydrophobic dyes such as thioflavin T or Congo red, as well as antibodies that bind to a common epitope.^7,8^

Understanding the mechanism of aggregation into amyloid fibrils requires knowledge of the structure of the amyloid precursor conformation, the nature and identity of any assembly intermediates, and the structure of the fully formed amyloid fibrils themselves. Over the last few years, the nature of the initial assembly-competent state for a number of amyloidogenic proteins has been elucidated using approaches spanning bioinformatics, peptide libraries, and nuclear magnetic resonance (NMR).^9–14^ In parallel, using cryo-electron microscopy, atomic force microscopy (AFM), solid-state NMR and X-ray crystallography, information is beginning to emerge about the conformational properties of the amyloid fibril itself.^15–20^ However, the question of how the polypeptide chain is wound into the generic cross-*β* structure of amyloid for fibrils formed from different proteins remains elusive.

*β*_2_-microglobulin (*β*_2_m) is a 99-residue protein (11 860.4 kDa) that forms the non-covalently bound light chain of the class I major histocompatibility complex (MHC-I). Native *β*_2_m has a seven-stranded *β*-sandwich structure, in which strands A, B, D, and E form one *β*-sheet, while strands C, F, and G form the second *β*-sheet (Fig. 1(a)). The two *β*-sheets are held together by a single disulphide bond that links Cys25 and Cys80 in strands B and F, respectively.^21^ Aggregation of primarily full-length, w ild-type *β*_2_m to form classical amyloid fibrils occurs in all patients who have been subjected to long-term haemodialysis, resulting in the disorder dialysis-related amyloidosis (reviewed in Radford *et al*.^22^). However, in addition to the intact wild-type protein, up to 25% of *β*_2_m extracted from fibrils *ex vivo* contains deletions of 6–18 residues from the N-terminal of the polypeptide chain.^23^

Despite the identification of *β*_2_m as a human amyloid protein more than 20 years ago,^24^ little is known in detail about the molecular mechanism of amyloid formation from *β*_2_m, or the structure of amyloid fibrils formed *in vitro* or *in vivo* from this protein. Recent studies have shown that partial or more complete unfolding of *β*_2_m is required for fibril formation to occur *in vitro*.^11,12,25^ NMR analysis of the partially unfolded states of *β*_2_m formed under acidic conditions, together with a number of other studies, have provided important clues into the structural rearrangements required for the normally highly soluble native protein to form amyloid-like fibrils *in vitro*.^12,25–27^ These studies have shown that partially unfolded *β*_2_m formed by acidification to pH 3.6 displays significant loss of structure in the regions corresponding to the native A and G terminal strands and considerable destabilisation of residues 20–85 (corresponding to the native B–F strands),^11^ while more complete acid denaturation, to form a collapsed species lacking native secondary structure but containing significant non-native structure involving interactions between hydrophobic and aromatic residues, occurs at pH 2.5.^12,27^ These species give rise to fibrils with different morphology. Thus, at low ionic strength at pH 2.5, fibrils of a straight morphology >μm in length with a defined helical period ^28,29^ are formed with nucleation-dependent kinetics.^30,31^ At pH 3.6, by contrast, short straight fibrils form at the same ionic strength.^29^ Finally, fibrils formed at pH 2.5 or 3.6 under high ionic strength conditions (i.e. in the presence of >200 mM sodium chloride) have a beaded or worm-like morphology.^25,28,29^

In order to define the region of *β*_2_m that forms the amyloidogenic core of fibrils with different morphology, a systematic and comprehensive study of the structural properties of *β*_2_m monomer and the fibrils formed under varying ionic strength and pH conditions has been undertaken using limited proteolysis in conjunction with electrospray ionisation tandem mass spectrometry (ESI-MS/MS). The enzyme of choice was pepsin due to its broad specificity at the acidic pHs employed to unfold *β*_2_m and to form amyloid-like fibrils. The propensity of pepsin to cleave at a wide range of amino acid residues^32^ leads to the possibility of over 30 theoretical cleavage sites spread throughout the 99-residue protein monomer and therefore provides the opportunity for comprehensive coverage of all regions of the protein. In addition, seven peptides that together constitute the entire 99 residues of *β*_2_m were digested both at pH 2.5 and 3.6 to provide a framework from which to compare the protease accessibility of the *β*_2_m monomer and fibrillar forms. The peptide digest fragments in all experiments were unambiguously identified from the molecular mass and sequence information obtained using ESI-MS(/MS).

These results provide the first detailed insights from a single, all-inclusive survey into the protected core regions in these different fibrillar types and show that, while all fibrillar species analysed contain amyloid-like properties, the packing of the polypeptide chain in fibrils with different structural morphologies varies significantly.

## Experimental

### Preparation of *β*_2_m and fib ril growth

*β*_2_m was over-expressed in *E. coli* and purified to homogeneity as described previously.^33^ An N-terminal initiating Met residue (Met0) was incorporated into ∼95% of *β*_2_m prepared by this method, as determined by ESI-MS. Fibrils were formed by incubating *β*_2_m freshly dissolved (1 mg mL^−1^) in buffer (25 mM sodium phosphate, 25 mM sodium acetate) either lacking NaCl (low ionic strength) or containing NaCl (final total ionic strength of 400 mM). Incubation was for 7 days at 37°C either with agitation (200 rpm) for samples at low ionic strength, or without agitation for samples in the presence of NaCl, as described previously.^33^

### Pepsin proteolysis

#### Limited proteolysis of monomeric β_2_m and peptides

For proteolysis experiments with monomeric *β*_2_m, an aqueous stock solution of native *β*_2_m (5 mg mL^−1^; pH 6 in unbuffered water) was prepared from purified lyophilised recombinant protein. The protein was diluted with water to a final protein concentration of 1 mg mL^−1^ and adjusted to a final pH of 2.5 or 3.6 with HCl. The sample was then incubated at ambient temperature for 20 min to allow the protein to populate the partially folded or acid-denatured states. Previous experiments have shown that under these conditions *β*_2_m remains entirely monomeric.^11,12^ Pepsin (Sigma, Poole, UK) (1:100 w/ w pepsin/ *β*_2_m) was added and proteolysis allowed to proceed at 25°C for either 15 min or 24 h. The reaction was inhibited by addition of pepstatin A (Sigma) (10 μM final concentration). Samples were then frozen on dry ice and stored at −20°C prior to ESI-MS analysis.

The peptide samples were subjected to pepsin proteolysis at a molar concentration of 83 μM (equivalent to 1 mg mL^−1^*β*_2_m) under the same conditions used for the intact protein. The peptides were obtained as described previously.^31^

#### Limited proteolysis of amyloid fibrils of β_2_m

For pepsin digestion of fibrillar samples, fibrils formed under different conditions (see above) were purified from residual soluble material by centrifugation (13 000 rpm, 10 min), resuspended with the original volume of buffer used to generate the fibrils, and the fibrils again collected by centrifugation. This procedure was repeated three times. The samples were finally resuspended with the original volume of buffer used to generate the fibrils. Pepsin (1:100, w/w relative to the concentration of *β*_2_m monomer in fibrillar form, determined by denaturation of a fibril sample with guanidinium chloride and calculation of the protein concentration using absorbance at 280 nm as described previously^34^) was added and digestion allowed to proceed for 15 min or for 24 h at 25°C. After 15 min, the fibrillar samples displayed very few cleavage sites (i.e. limited proteolysis was observed). The digests were also allowed to proceed for 24 h in order to reveal all possible cleavage sites (i.e. extensive proteolysis). The reaction was then quenched by treating the digested sample with pepstatin A as described above. The digested fibrils were collected by centrifugation and the pellet resuspended in Milli-Q water adjusted to pH 7 with NaOH. Incubation at this pH overnight with agitation resulted in extensive depolymerisation of the sample as demonstrated by light scattering, negative stain electron microscopy and thioflavin T fluorescence.^35^ Gel filtration analysis of the resulting samples carried out by elution of the sample through a Superose 6 column (molecular weight range 10–100 kDa; Amersham Biosciences, Little Chalfont, UK) with aqueous NaOH (pH 7), using bovine serum albumin (66 kDa) and carbonic anhydrase (29 kDa) as markers, verified (absorbance at 280 nm) that >70% *β*_2_m had reverted to a monomeric state after fibril depolymerisation. The resulting material was stored at −20°C prior to ESI-MS analysis.

### AFM imaging of b_2_m samples

Samples were imaged immediately or after rapid freezing on dry ice followed by storage at −20°C. Aliquots (10 μL) of each sample were deposited onto freshly cleaved mica (Agar Scientific, Stansted, UK) and incubated for 1 min. The surface was then rinsed with deionised water (10 mL) and dried with compressed nitrogen (1 bar). Samples were imaged by tapping mode AFM in air using a Nanoscope III Multimode atomic force microscope (Veeco Instruments Inc., Santa Barbara, CA, USA) equipped with an E-Scanner (scan range ∼13 μm). Rectangular silicon cantilevers (160 μm Long) with resonance frequencies in the range of 232–311 kHz and nominal spring constants in the range 13–103 N m^−1^ (typically 42 N m^−1^) (Olympus Corp., Tokyo, Japan) were excited slightly below the free resonance frequency to ensure that the microscope was operating in repulsive force regime. The cantilevers have integrated tips with a radius of curvature <10 nm and a tip opening angle of 35°. Samples were imaged at scan sizes between 1 and 3 μm using line scan rates below 2 Hz and (512 × 512) pixels were collected per image. Images were flattened using a second-order planefit in the Nanoscope image analysis software.

### ESI-MS/MS analysis of proteolytic digests

ESI-MS/MS analyses were performed on a Q-Tof 1 quadrupole time-of-flight (TOF) mass spectrometer equipped with nano-ESI and a capillary high-performance liquid chromatography (HPLC) system (Waters Corp., Manchester, UK). Positive ionisation was used for the sample analyses and an external calibration using horse heart myoglobin was applied. The MassLynx software supplied by Waters Corp. was used for data processing. For the monomeric protein and peptide samples, an aliquot (2–3 μL) was placed directly into a gold-plated, borosilicate capillary and inserted into the ionisation source. A capillary voltage of 900 V and a sampling cone voltage of 40 V were set. Depolymerised samples were analysed by on-line capillary HPLC/ESI-MS/MS using a reversed-phase C18 column (75 μm i.d. 15 cm; LC Packings, Amsterdam, The Netherlands) with a gradient of 5–80% aqueous acetonitrile with a constant 0.1% formic acid added over 40 min flowing at 1 μL min ^−1^ with a back pressure of ∼1150 psi. A sample injection of 1 μL was made. A capillary voltage of 3 kV and a sampling cone voltage of 40V were set for these analyses. The capillary HPLC/ ESI-MS/MS process thus had the dual effect of desalting the samples and achieving peptide fragmentation.

To generate a molecular mass map, the quadrupole analyser was used in wide band-pass mode and the TOF analyser used for the *m/z* analysis. Spectra were acquired over the appropriate *m/z* range with an accuracy of ±0.1 Th. To generate sequence data for the peptides, MS/ MS experiments were carried out whereby the quadrupole analyser was used to select individually the ions of interest (± 4 Th). Argon was admitted into the collision cell so that the pressure in the cell increased by a factor of 10 and the collision energy set in the range 10–60 eV. The resulting product ions were analysed by the TOF analyser. MS/MS analyses were performed manually for those samples analysed directly from borosilicate vials and automatically for samples requiring capillary HPLC/MS analysis.

### ESI-MS determination of *β*_2_m monomer concentration in fibrillar samples

To determine whether the fibrils were stable during the incubation time with pepsin, the concentration of monomer in the solution of purified fibrils was determined using online HPLC/MS (HP1100, Agilent Technologies, Cheadle, UK) with a Platform II quadrupole mass spectrometer (Waters Corp.). A calibration curve was constructed to allow *β*_2_m quantification by analysing samples containing 16.8, 37.6, 54.4, and 83 μM *β*_2_m dissolved in Milli-Q water. The samples were chromatographed on a reversed-phase HPLC column (2.1 mm i.d. × 20 cm; Vydac, Hesperia, CA, USA) with a solvent gradient of 0–70% aqueous acetonitrile with a constant 0.1% formic acid added over 20 min at a flow rate of 200 μL min^−1^. An ESI capillary voltage of 3.5 kV, a sampling cone voltage of 40 V, and a source temperature of 90°C were set. The peak areas from the resulting chromatograms were integrated and a calibration curve drawn which was used to determine the quantity of *β*_2_m monomer released from the fibrils during digestion with pepsin. For these experiments, fibrils formed in buffer (25 mM sodium phosphate, 25 mM sodium acetate) at pH 2.5 and 3.6, either in the presence or absence of additional NaCl, were washed by centrifugation at 13 000 rpm for 10 min (×3). The final pellet was suspended in the original buffer and, at 0, 15, and 30 min time points, each sample was analysed by reversed-phase HPLC/MS under the same conditions as the calibration samples. None of the fibril samples showed a detectable monomer peak, demonstrating that the fibrils remained stably assembled throughout the digest.

## Results

### Comparison of the morphology of *β*_2_m monomer and fibrillar species by AFM

In order to confirm the morphological structures of the fibrils formed from *β*_2_m at pH 2.5 and 3.6 (under both low and high ionic strength conditions), the samples were imaged by AFM (tapping mode) prior to a structural investigation by limited proteolysis. AFM tapping mode allows high-resolution imaging of delicate sample surfaces by placing the oscillating tip in contact with the surface to provide a high-resolution image and then lifting the tip off the surface for repositioning before taking a subsequent image, rather than dragging the tip across the surface.

**Figure 1:**
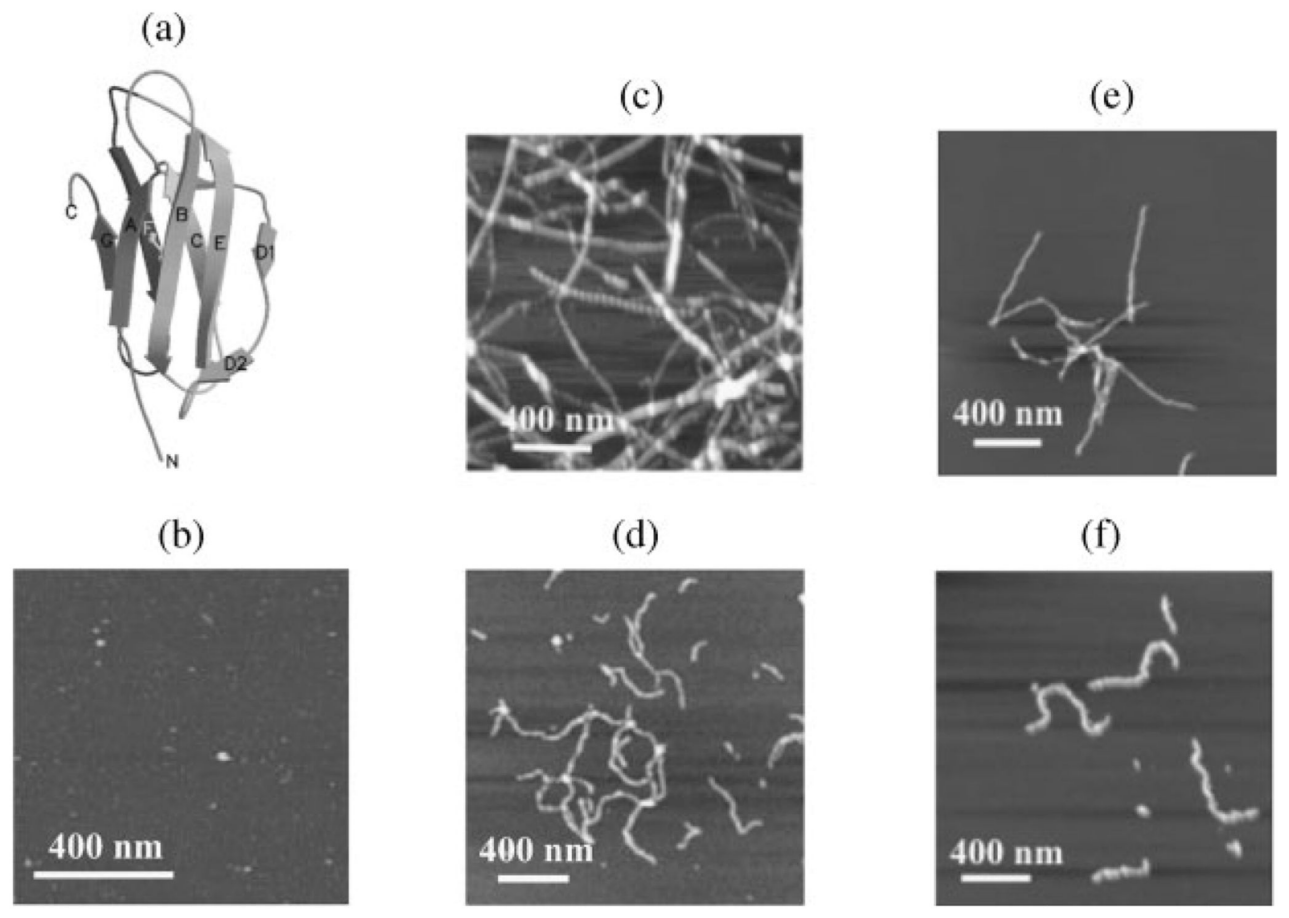
(a) Ribbon diagram of native *β*_2_m showing the seven *β*-strands (labelled A to G) and the disulphide bond between strands B and F (linking Cys25 and Cys80). The figure was drawn using Molscript^47^ and Raster3D^48^ using the coordinates 1DUZ^21^. (b–f) Tapping-mode AFM images showing: (b) monomeric *β*_2_m (pH 7.0); (c–f) fibrils formed by incubating *β*_2_m at: (c) pH 2.5 (low ionic strength); (d) pH 2.5 (high ionic strength); (e) pH 3.6 (low ionic strength); and (f) pH 3.6 (high ionic strength).

The resulting AFM images are shown in Figs. 1(b)–1(f). As expected, the AFM image of monomeric *β*_2_m at pH 7 shows a layer of monodisperse globular particles with an average diameter of ∼10 nm, consistent with monomeric *β*_2_m broadened by the AFM tip (Fig. 1(b)). Previous studies using ultracentrifugation and pulsed-field gradient NMR experiments have also shown that *β*_2_m remains monomeric at both pH 2.5 and 3.6 in 100% aqueous solvent, while the addition of buffer salts facilitates the formation of dimers and trimers over a 20-min incubation period.^33^

The long, straight fibrils shown in Fig. 1(c) are the end point of fibril formation from acid-unfolded, monomeric *β*_2_m at pH 2.5 under low ionic strength conditions. These fibrils extend to μm lengths and are long and straight with a twisted helical period. Consistent with previous results, a range of fibril subtypes is observed that vary both in the number of constituent protofibrils and in their helical period.^28,33^ By contrast, fibrils grown from partially unfolded, monomeric *β*_2_m at pH 3.6 under low ionic strength conditions, and also with agitation, are apparently rigid in appearance but in general less than ∼500 nm in length, consistent with previous results^29^ (Fig. 1(e)). However, when *β*_2_m is incubated at pH 2.5 or 3.6 under high ionic strength conditions and in the absence of agitation, the resulting worm-like fibrils have a typical curved and nodular appearance that is generally shorter than ∼600 nm in length (Figs. 1(d) and 1(f), respectively).

### Probing the structural properties of monomeric and fibrillar *β*_2_m using limited proteolysis and ESI-MS/MS

#### Determination of the efficacy of pepsin digestion using peptide models of β_*2*_m

**Figure 2:**
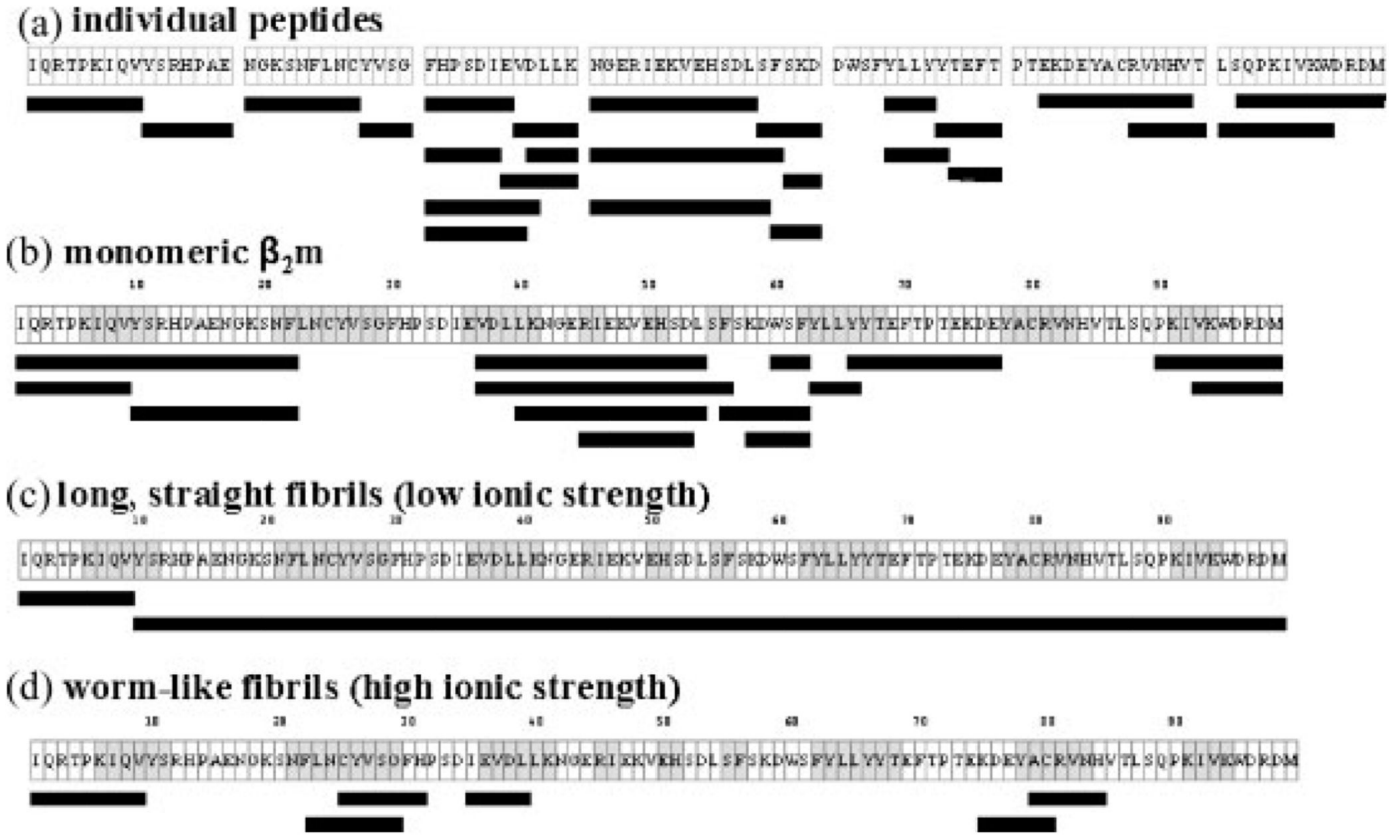
Limited pepsin proteolysis at pH 2.5 of (a) seven synthetic peptides which together comprise the entire sequence of *β*_2_m; (b) monomeric *β*_2_m; (c) fibrils formed under low ionic strength conditions; and (d) fibrils formed under high ionic strength conditions. The 99-residue amino acid sequence is shown in (b)–(d) (without the N-terminal initiating Met residue). The regions of grey shading indicate the location of the native *β*-strands. Black bars under the sequences show the peptide fragments identified by ESI-MS(/MS) after limited proteolysis.

**Figure 3:**
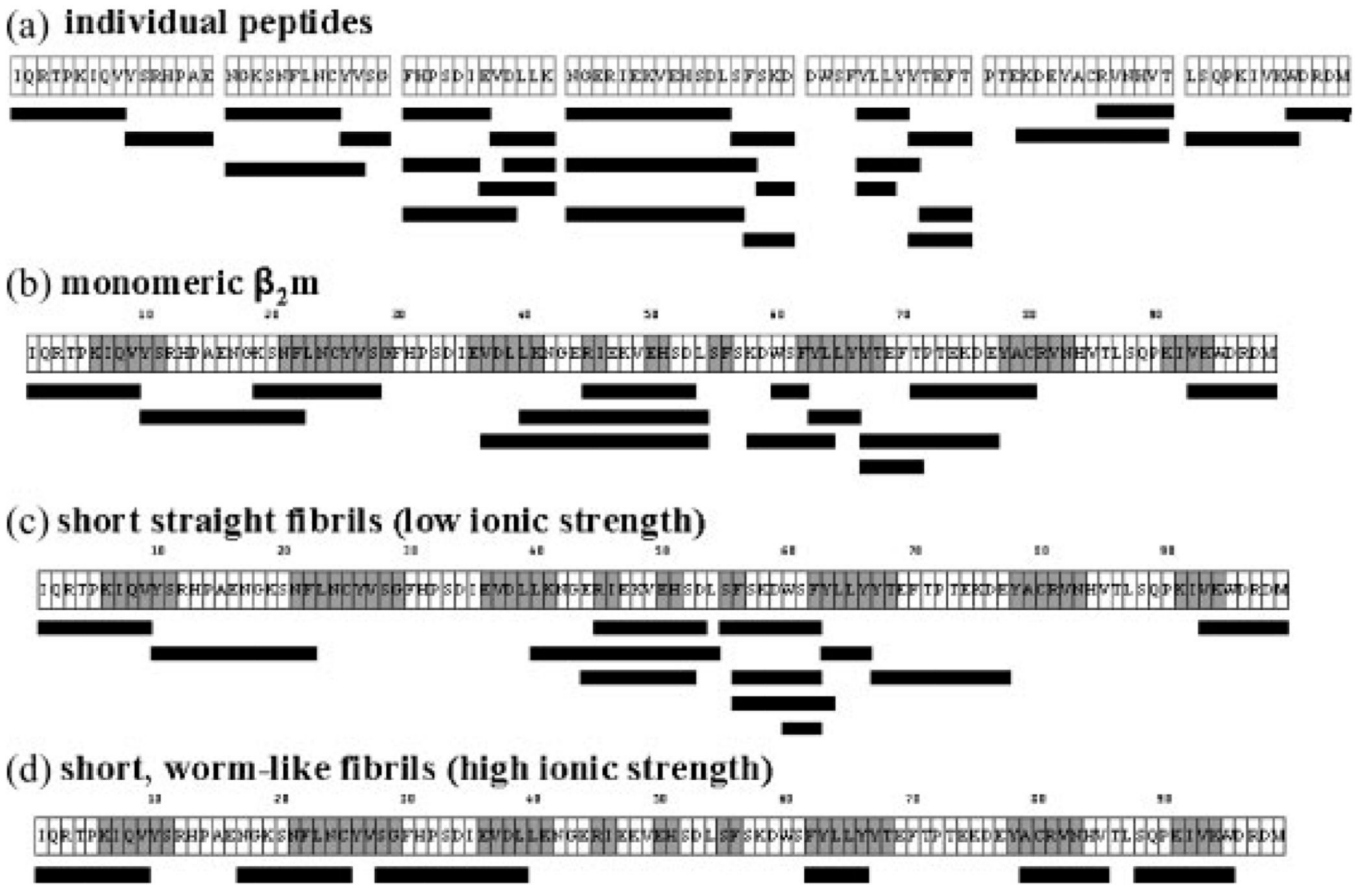
Limited pepsin proteolysis at pH 3.6 of (a) seven synthetic peptides which together comprise the entire sequence of *β*_2_m; (b) monomeric *β*_2_m; (c) fibrils formed under low ionic strength conditions; and (d) fibrils formed under high ionic strength conditions. The 99-residue amino acid sequence is shown in (b)–(d) (without the N-terminal initiating Met residue). The regions of grey shading indicate the location of *β*-strands. Black bars under the sequences show the peptide fragments identified by ESI-MS(/MS) after limited proteolysis.

Pepsin was the enzyme of choice for these limited proteolysis experiments because of its broad specificity under the pH conditions required *in vitro* for *β*_2_m amyloid fibril formation. To map the cleavage sites for pepsin in an unstructured peptide of the sequence of *β*_2_m, seven synthetic peptides, which together comprise the entire seven *β*-strands and the connecting loops of intact *β*_2_m, were synthesised and subjected to pepsin digestion at both pH 3.6 and 2.5. The seven peptides comprised the following amino acid residues of the intact protein: 1–16, 17–29, 30–41, 42–59, 59–71, 72–86, and 87–99, corresponding to regions of the polypeptide chain that encompass the native *β*-strands A, B, C, D, E, F, and G, respectively, together with several residues from the preceding and succeeding loops and turns (Figs. 1(a), 2(a) and 3(a)). The two disulphide-bond forming cysteines (Cys25 and Cys80) were substituted with serine residues to avoid any oxidative peptide dimerisation. The peptide containing residues 59–71 was found to undergo fibrillisation spontaneously^31^ under all pH conditions and hence proteolysis in this case was performed on a mixture of the peptide and any co-existing higher order aggregates. All other peptides showed no evidence of self-association into fibrillar structures under the conditions used.

Pepsin digestion was carried out at pH 2.5 or 3.6 under both limited (15 min) and extensive (24 h) proteolysis time-scales for each synthetic peptide. The digest mixtures were analysed by ESI-MS initially, to generate molecular masses for all of the components present, and then by ESI-MS/MS to generate sequence information for each peptide fragment. Due to the broad substrate specificity of pepsin, a molecular mass alone was often insufficient to identify the peptide fragments uniquely and hence sequence information was required. Pepsin cleavage sites were found throughout the synthetic peptides after 15 min, as expected from the unstructured nature of the peptides, with little difference between the cleavage sites observed at pH 2.5 and 3.6 (Figs. 2(a) and 3(a)). The peptide fragments detected from the limited digestion were not significantly different from those obtained after extensive digestion (data not shown), consistent with rapid cleavage of the peptides under these experimental conditions. The data thus confirm the susceptibility of unfolded *β*_2_m to digestion by pepsin throughout its sequence at precisely the pHs of *in vitro* fibril formation.

#### Pepsin proteolysis of monomeric β_*2*_m

ESI-MS, circular dichroism (CD) and fluorescence spectroscopy studies have led to the conclusion that monomeric *β*_2_m is partially unfolded at pH 3.6,^11,25,36^ while, at pH 2.5, NMR studies have shown acid-unfolded, monomeric *β*_2_m to be largely disordered, although some residual structure is retained, in part due to the Cys25-Cys80 disulphide bridge (which links the native strands B–F).^12,27^ To determine the influence of the residual structure of the unfolded, intact protein on the susceptibility to pepsin cleavage, both limited and extensive pepsin proteolyses were carried out on monomeric *β*_2_m at pH 2.5 and 3.6 under identical conditions to those used above for the individual peptides.

**Figure 4:**
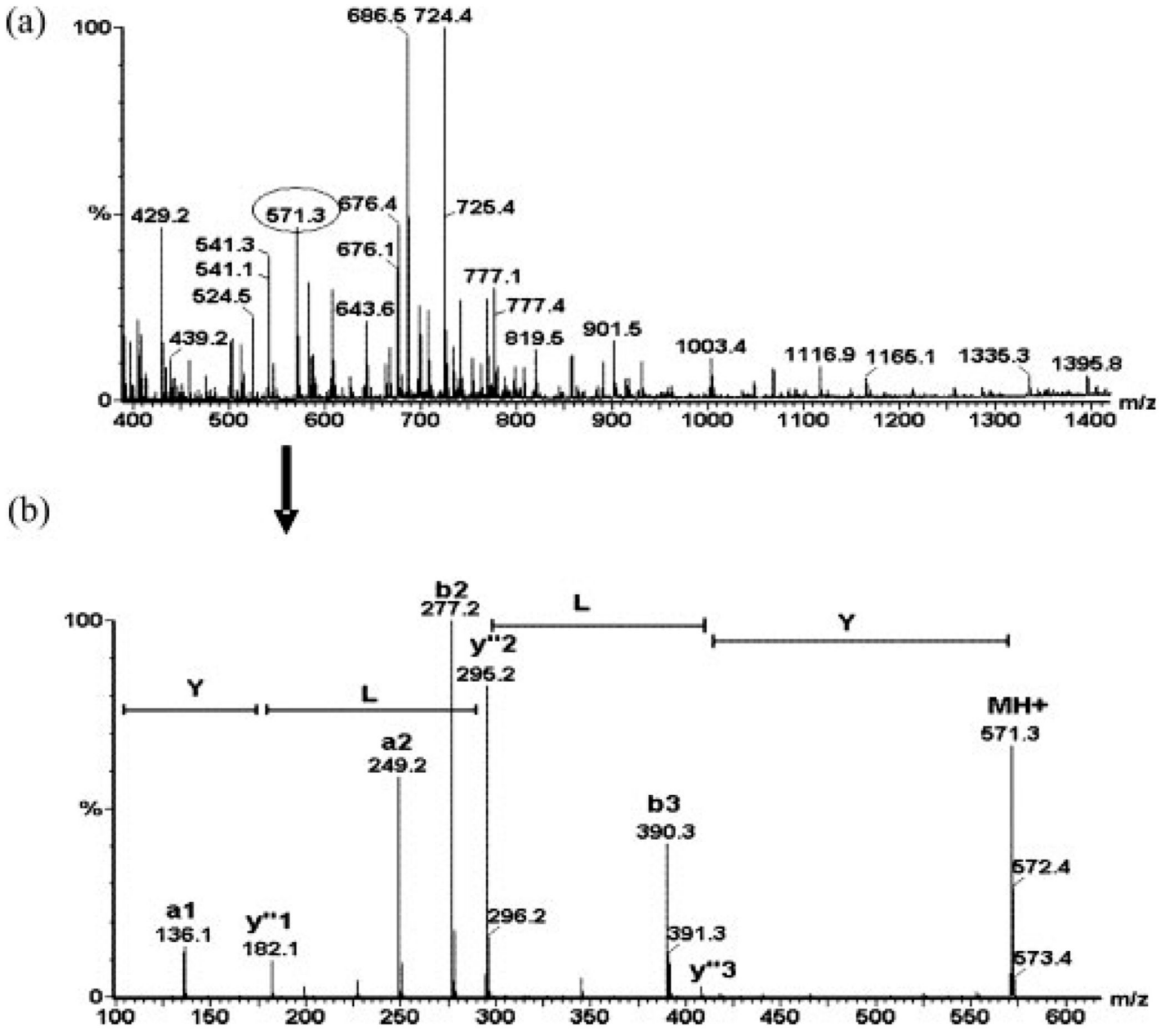
(a) ESI-MS mass spectrum of monomeric *β*_2_m after pepsin proteolysis at pH 2.5. The protein was incubated with pepsin (100:1 w/w *β*_2_m/pepsin) at 258C for 15 min and the resulting mixture analysed on a Q-Tof mass spectrometer (Waters Corp., Manchester, UK) equipped with a nano-ESI source. The spectrum shows a mixture of singly and multiply charged protonated molecules. The MH^+^ ion at m/z 571.3 has been circled as an example of a peptide that was subsequently subjected to MS/MS analysis (see (b)). (b) MS/MS analysis of the MH^+^ ion at m/z 571.3 in (a). MS/MS analyses were performed by fragmentation of the selected precursor ions in the collision cell. The product ions were analysed and the resulting spectra interpreted for sequence information. The a, b, and y″ sequence-specific ions labelled on the spectrum confirm the sequence of Tyr.Leu.Leu.Tyr (residues 63–66; 570.3 Da).

The ESI-MS spectra generated from the pepsin digestion of intact *β*_2_m were complex, as illustrated in Fig. 4(a), which shows the mass spectrum obtained from proteolysis of *β*_2_m monomer at pH 2.5. The molecular masses of the peptide components were measured, and each component <2500 Da in mass was further characterised by MS/MS. For example, Fig. 4(b) highlights the MS/MS fragmentation spectrum from the MH^+^ ions at *m/z* 571.3 (circled in Fig. 4(a)). The a, b, and y″ sequence-specific ions labelled on the MS/MS spectrum confirm the sequence of Tyr.Leu.Leu.Tyr (residues 63–66) thus characterising this peptide unambiguously and distinguishing it from the isobaric possibility Leu.Leu.Tyr.Tyr (residues 64–67). In many cases MS/MS analysis was necessary to provide unambiguous identification of the peptides.

**Figure 5:**
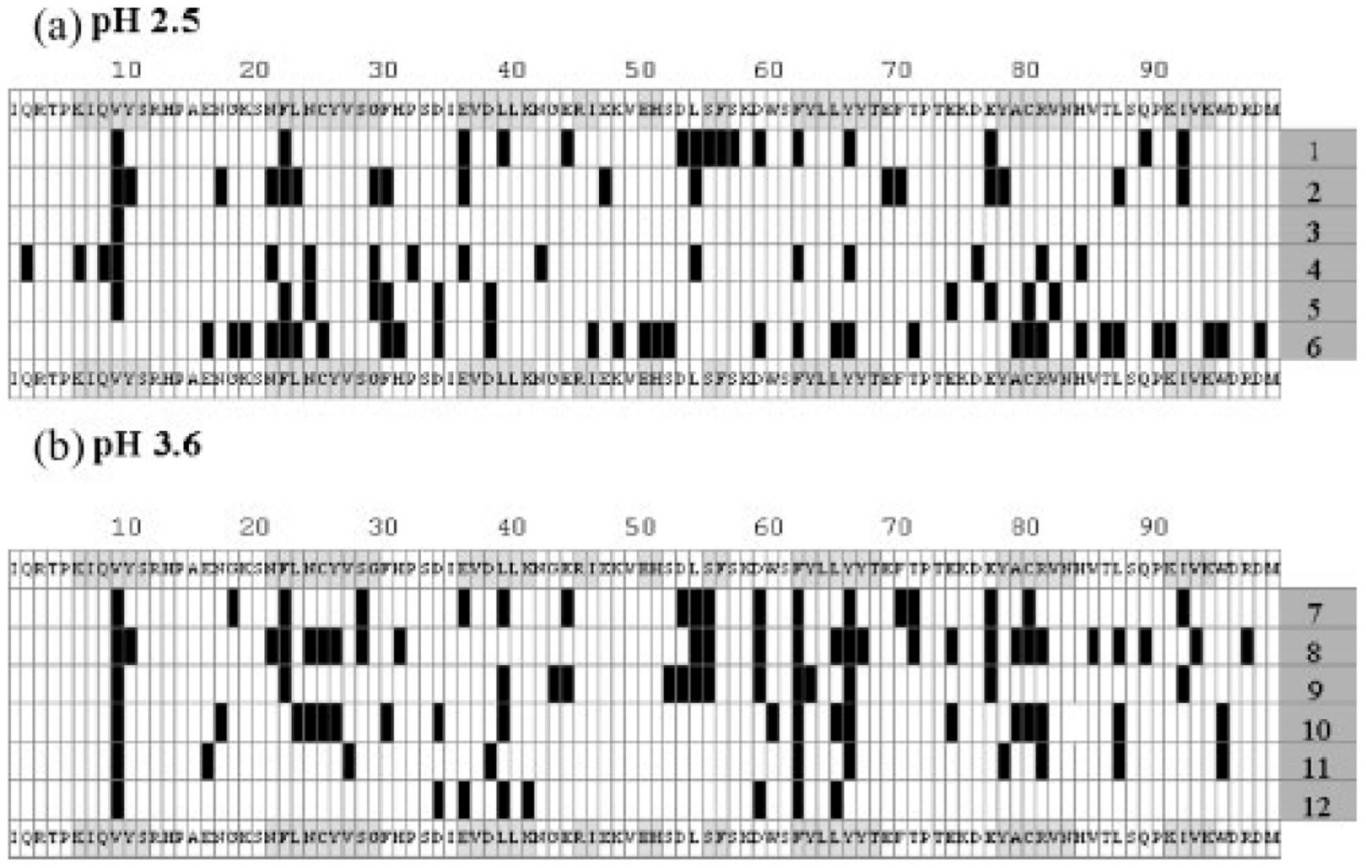
Summary of the cleavage sites observed by ESI-MS(/MS) after pepsin proteolysis. The rows illustrate data obtained at (a) pH 2.5: (1) and (2) monomeric *β*_2_m (15 min and 24 h digestion, respectively), (3) and (4) fibrils formed under low ionic strength conditions (15 min and 24 h digestion, respectively), and (5) and (6) fibrils formed under high ionic strength conditions (15 min and 24 h digestion, respectively); (b) pH 3.6: (7) and (8) monomeric *β*_2_m (15 min and 24 h digestion, respectively), (9) and (10) fibrils formed under low ionic strength conditions (15 min and 24 h digestion), and (11) and (12) fibrils formed under high ionic strength conditions (15 min and 24 h digestion, respectively). Limited proteolysis was performed with pepsin/*β*_2_m (1:100 w/w) at 258C for 15 min, and extensive proteolysis for 24 h. The 99-residue amino acid sequence is shown (without the N-terminal initiating Met residue). The regions of grey shading indicate the location of *β*-strands. The vertical bars in (a) and (b) depict the C-terminal residue of an identified peptide fragment and hence indicate the proteolytic cleavage sites.

The proteolytic fragments identified in the limited digests of monomeric *β*_2_m at both pH 2.5 and 3.6 were broadly similar (Figs. 2(b) and 3(b), respectively). These digestion patterns also exhibited a strong similarity to those obtained from the proteolysis of the seven individual peptides (compare with Figs. 2(a) and 3(a)) in that cleavage sites were revealed throughout the protein sequence in the regions corresponding to all seven native *β*-strands and their connecting loops. Extensive proteolysis gave similar cleavage maps, although some of the peptide fragments detected after 15 min had been further proteolysed and were no longer detected after 24 h (Figs. 5(a) rows 1–2, and 5(b) rows 7–8). The results demonstrate, therefore, that monomeric *β*_2_m displays little protease resistance at both pH 2.5 and 3.6, consistent with the results of NMR^11,12,27^ and CD,^25^ indicating that the protein adopts non-native and highly dynamic structures at these pH values.

#### Comparison of β_*2*_m amyloid fibrils of different morphologies

Fibrils with different morphological properties formed at pH 2.5 and 3.6 under both low and high ionic strength conditions were also subjected to limited and extensive pepsin proteolysis under identical conditions to those described above. The proteolysis times were optimised to 15 min (limited proteolysis) and 24 h (extensive proteolysis) using the different fibril types. Extreme care was taken to ensure that the fibrils were separated from any lower order aggregates before proteolysis and that after pepsin treatment the vast majority of fibrillar material was depolymerised to monomer before MS analysis (see Experimental). Additionally, to confirm the stability of the fibrils during proteolysis, the equilibrium concentration of monomeric *β*_2_m released during the 15 min digest was shown to be negligible (see Experimental). The limited proteolysis maps from these four fibrillar species are detailed in Figs. 2(c), 2(d), 3(c) and 3(d).

For the long, straight fibrils formed at pH 2.5 under low ionic strength conditions (Fig. 1(c)), limited pepsin proteolysis produced the dramatic result that only a single cleavage site at Val9 was observed; the remainder of the polypeptide chain (residues 10–99) remained intact indicating that this region is incorporated into the fibrillar structure where it is protected from cleavage (Fig. 2(c)). Importantly, however, extensive proteolysis of these fibrils for a digestion time of 24 h resulted in further cleavage sites throughout the protein sequence, suggesting either that other sites show limited susceptibility to pepsin, or that transient fibril dissociation followed by rapid reassociation into the fibril^37^ over the longer time period of digestion results in the cleavage sites observed (Fig. 5(a), rows 3–4). By contrast with these results, the shorter, worm-like fibrils formed at pH 2.5 under high ionic strength conditions (Fig. 1(d)) showed a very different pattern of resistance to pepsin proteolysis (Fig. 2(d)). New cleavage sites were detected, predominantly in regions corresponding to the native strands B, C and F, together with the common cut-site at Val9. Significantly, however, few cleavage sites were observed between residues 40 and 74, in marked contrast with the results obtained from the peptide models and the monomeric protein under identical conditions (Figs. 2(a) and (b)), suggesting that these residues form a protective core in fibrils of this type. Again, extensive proteolysis yielded cleavage sites throughout the protein sequence (Fig. 5(a), rows 5–6).

The fibrils formed at pH 3.6 under low ionic strength conditions, which have a short, straight morphology (Fig. 1(e)), showed a similar pattern of pepsin proteolysis to those determined for the individual peptides and monomeric *β*_2_m, with little evidence of protection from proteolytic cleavage in one particular region (Fig. 3(c)). These data suggest that these fibrils do not possess a highly structured core. Extensive proteolysis gave similar results; again some of the peptides detected after 15 min had been digested further and hence were not visible after 24 h (Fig. 5(b), rows 9–10). By contrast with these results, the worm-like fibrils formed at the same pH, but at high ionic strength, showed many fewer cleavage sites, suggesting that the region 40–61 forms the core of this fibril type (Figs. 3(d) and 5(b), rows 11–12).

## Discussion

Figure 5 summarises the complete data sets from the limited and extensive pepsin proteolysis experiments performed at pH 2.5 and 3.6 on monomeric *β*_2_m and the fibrils formed under both low ionic and high ionic strength conditions. Previously, at pH 2.5, monomeric *β*_2_m has been found to be acid-unfolded and highly unstructured, retaining only residual non-native hydrophobic clusters in the region of residues 25–80.^12,27^ At pH 3.6, however, the protein has been found to be partially unfolded retaining stable structure in regions that correspond to the native *β*-strands B, C, D, E and F, while the ten N-terminal residues are dynamic.^11^ Here we have shown, using limited proteolysis, that monomeric *β*_2_m is highly susceptible to proteolytic digestion throughout the entire sequence at both pH 2.5 and 3.6, confirming that a structure persistent enough to protect the protein against proteolysis does not remain at these pH values. The proteolytic cleavages observed at both pH 2.5 and 3.6 in the N- and C-terminal regions of the protein (Figs. 2(b), 3(b), 5(a) and 5(b)) are of significant interest, as removal of the six N-terminal residues of *β*_2_m has been shown to increase the ability of the protein to form fibrils in seeded reactions at neutral pH.^38^ It has also been suggested that strands A and G are flexible within the native structure and that local unfolding of one or more of these strands initiates fibril formation at pH 7.^11,39,40^ Additionally, NMR studies have shown that the first ∼20 residues of the polypeptide chain and ∼15 residues of the C-terminal region of acid-unfolded *β*_2_m at pH 2.5 are highly dynamic and closely represent a random coil,^12^ consistent with the observation that several residues in these regions are cleaved by pepsin at this pH. However, the central region of monomeric *β*_2_m, encompassing residues 25–80 which contains the Cys25-Cys80 disulphide bond, has been found to contain significant residual structure measured using NMR relaxation methods.^12^ This region, which is particularly rich in aromatic residues that are especially prone to cleavage with pepsin, was found to be highly susceptible to limited proteolytic cleavage at both pH 2.5 and 3.6, indicating that any residual structure is insufficient to protect these residues from proteolysis, concurring with the conclusion that acid-unfolded, monomeric *β*_2_m is a rapidly fluctuating species.^12^

Significantly, limited proteolysis of the long, straight fibrils formed at pH 2.5 under low ionic strength conditions showed only a single cleavage site at Val9 (Fig. 2(c)), indicating that the remainder of the unfolded monomer (residues 10–99) becomes tightly folded and/or sequestered from solvent in the fibril structure such that it is highly protected from enzyme cleavage. By contrast, the N-terminal ∼10 residues remain dynamic and are rapidly cleaved by the protease, consistent with results from others that show this region of the polypeptide chain to be solvent accessible in the fibrillar state.^41,42^ The C-terminal region, however, is incorporated into this fibrillar structure. Recent NMR data on fibrils formed at pH 2.5 have suggested that the C-terminal region is more protected from hydrogen exchange than the N-terminal in the fibrillar state, but less protected than the remainder of the polypeptide chain, and that, for the majority of molecules, the C-terminal forms an integral part of the *β*-sheet core.^41,42^ Interestingly, by contrast with the results of proteolysis of similar fibrils using a range of proteases,^23,43^ no cleavage of the C-terminal region was observed for the fibrils under the conditions employed here. Hence the presence of a single cut-site at Val9 indicates that the fibrils formed at pH 2.5 are highly structured throughout the region 10–99, and the acid-unfolded *β*_2_m monomer, therefore, must undergo significant refolding during fibrillisation.

The limited proteolysis cleavage properties of the worm-like fibrils formed at pH 2.5 under high ionic strength conditions (Fig. 2(d)) differ significantly from those of the long, straight fibrils formed at the same pH. For this fibril type, residues in the central region of the polypeptide chain (40–74) are protected from proteolysis, whilst the remainder of the polypeptide chain is readily cleaved, implying that a hydrophobic core of only ∼35 residues forms an integral part of the fibril structure. The short, worm-like fibrils, therefore, are less tightly organised compared with their long, straight counterparts, again consistent with hydrogen-exchange experiments on similar fibrils that are much less protected from exchange.^42^

Proteolysis of the fibrils formed from partially unfolded, monomeric *β*_2_m at pH 3.6 gave further insights into fibrillar morphologies. Under low ionic strength conditions, the short, straight fibrils formed proved to be highly susceptible to limited proteolysis (Fig. 3(c)), showing a digestion pattern similar to that of monomeric *β*_2_m at this pH. However, the worm-like fibrils formed under high ionic strength conditions were less susceptible to limited proteolysis, indicating a protected core encompassing residues 40–61, similar to the fibrils with similar morphology formed at lower pH (Fig. 3(d)). Proteolysis of the individual peptide whose sequence covered this region led to cleavage at residues Leu54, Ser55 and Phe56, and the corresponding region in monomeric *β*_2_m is also susceptible to cleavage, suggesting that the lack of proteolysis is not due to a deficiency of protease specificity, but rather indicates a protected region. Fibrils formed under both high and low ionic strength conditions at pH 3.6 were susceptible to proteolytic cleavage in the N- and C-terminal regions, consistent with the observations that destabilisation of these regions has been shown to be important in fibril formation at this pH,^11,34^ and also that fibrils formed at pH 4 followed by proteolysis at pH 7 showed cut-sites in both the C- and N-terminal regions (Lys6 and Lys91).^23,43^

## Conclusions

Significant insights into the structural characteristics of amyloid fibrils have been deduced from detailed investigation using limited proteolysis.^23,43–46^ The ESI-MS/ MS data presented here illustrate clear differences in the fibrils formed from partially unfolded and acid-unfolded monomeric *β*_2_m which have different morphological properties, yet contain an architecture consistent with the common cross-*β* configuration of amyloid.^35^ The long, straight fibrils formed at pH 2.5 show dramatically enhanced protection from limited proteolysis compared with fibrils formed at pH 3.6. The data show that ∼90% of the polypeptide chain is involved in the final fibrillar structure at pH 2.5, while the protected core is reduced from ∼90 to ∼30 residues in fibrils with a worm-like morphology, independent of the pH at which the fibrils are formed. The limited proteolysis results also indicate that, while residues close to the Cys25-Cys80 disulphide bond are involved in the fibrillar structures formed at pH 2.5 under low ionic strength conditions, these residues are more susceptible to proteolysis in fibrils formed at pH 3.6 under high ionic strength conditions. Finally, the N-terminal ∼10 residues are highly susceptible to proteolytic cleavage in all fibril forms studied, in contrast to the C-terminal region which forms part of the protected core of the fibrils formed at pH 2.5, but is susceptible to proteolysis in the fibrils formed at pH 3.6.
